# Recurrent multifocal osteomyelitis (CRMO); effect of neridronate

**DOI:** 10.1186/1546-0096-12-S1-P260

**Published:** 2014-09-17

**Authors:** Federica Fontana, Chiara Forni, Nadia Scotti, Laura Timpone, Francesca Gicchino, Maria Alessio

**Affiliations:** 1 Department Of Pediatrics Federico Ii University, Naples, Italy; 2 Department of Pediatrics, Second University of Naples, Naples, Italy

## Introduction

Chronic recurrent multifocal osteomyelitis (CRMO) is an autoinflammatory bone disease of unknown etiology. Clinically, the disease is characterized by the insidious onset of local pain and swelling in affected bones. Its course is one of intermittent periods of exacerbation and remission with successive bones affected.CRMO most commonly affects the metaphysis of long bones, especially the tibia, femur, and clavicle. The spine, pelvis, ribs, sternum, and mandible may also be affected. Although lesions are mostly multiple, patients may present with a single symptomatic focus. Treatment in CRMO is empiric, since placebo controlled randomized trials have not been performed.

## Objectives

To describe the outcome of CRMO patients treated with neridronate.

## Methods

We report 8 patients (3 M.5 F, mean age 8ys) Median age of first CRMO symptoms was 6.3 years (range 5-13). The more affected sites were the metaphysis of the long bones, pelvis and coxofemoral joints.

## Results

Seven patients failed to respond to NSAIDs therapy. Two patients received corticosteroids, without clinical disease remission. Four patients received neridronate (2mg/kg body weight every 3 months for 1 year), all with good clinical response and induction of clinical remission. After a median follow-up period of 3.2 years (range 1-5), three patients are clinically asymptomatic and one patient required another 6 months course to reach and sustain remission.

## Conclusion

The treatment of CRMO is not standardized. Bisphosphonate therapy can be of benefit to patients with relapsing symptoms. Randomized controlled multicentric trials are needed to provide better evidence for definition of bisphosphonate therapy protocol.

## Disclosure of interest

None declared.

